# Innovations in mandibular advancement splint therapy for obstructive sleep apnoea

**DOI:** 10.3389/frsle.2023.1144327

**Published:** 2023-03-23

**Authors:** Anna Mohammadieh, Benjamin Tong, Philip De Chazal, Peter A. Cistulli

**Affiliations:** ^1^Department of Respiratory and Sleep Medicine, Royal North Shore Hospital, Sydney, NSW, Australia; ^2^Charles Perkins Centre, Faculty of Medicine and Health, University of Sydney, Sydney, NSW, Australia

**Keywords:** oral appliance, mandibular advancement splint, mandibular advancement device, sleep apnoea, obstructive sleep apnoea

## Abstract

Mandibular advancement splint (MAS) therapy emerged as an effective therapy for obstructive sleep apnoea (OSA) in the mid 1990s, and is now the leading treatment alternative for OSA. Since its inception, the field has seen a suite of revisions and advances in relation to design and customisation, fabrication, titration methods, response prediction models and the integration of data collection technology. This paper reviews these current and emerging innovations in MAS therapy and their impact upon sleep apnoea management.

## 1. Background: Mechanism of action and treatment response

MAS is an oral appliance which protrudes the mandible in relation to the maxilla, causing movement of soft tissues (tongue and soft palate) to increase the calibre of the upper airway and reduce its collapsibility. Although continuous positive airway pressure (CPAP) therapy is more effective than MAS at lowering the apnoea hypopnoea index (AHI) (Luz et al., 2022), CPAP acceptance and compliance rates may be low, leading to reduced overall efficacy in eliminating the burden of OSA (Grote et al., 2000; Schwartz et al., 2018). MAS therapy improves blood pressure, daytime somnolence, driving risk and quality of life to the same extent as CPAP, including in patients with severe OSA (Lim et al., 2006; Phillips et al., 2013). Therefore, it is hypothesised that the reduced efficacy of MAS when compared to CPAP may be offset by improved patient tolerance and adherence with MAS therapy (Schwartz et al., 2018), leading to similar benefits in neuro-behavioural and cardiovascular outcomes. However, one of the key barriers to wider uptake has been the variability of patient response, since up to 70% of patients will experience a partial or complete treatment response (Sutherland et al., 2015), leaving around 30% without a beneficial therapeutic outcome. In addition, since a MAS device relies on dental adherence in order to remain *in situ*, patients with inadequate dentition are ineligible and have been excluded from research studies. Prediction tools for a favourable MAS treatment response are an area of ongoing research.

## 2. Patient selection and prediction of response: Endotypes

Traditionally, demographic and anthropometric characteristics have been explored as markers of MAS treatment response. For example, younger age, less obesity, female gender, milder OSA, and supine-dependent OSA have variously been associated with treatment success, though these have all been weakly predictive (Sutherland et al., 2015). Endotypic profiling has been gaining recognition in Sleep Medicine generally as a means by which to advance precision medicine for OSA patients, and can be applied to MAS treatment response.

An endotype refers to a disease subtype with a distinct functional or patho-biological mechanism (Edwards et al., 2019). OSA endotypes include: arousal threshold (degree of ventilatory drive required to trigger an arousal from sleep), loop gain (instability in ventilatory control in response to a disturbance), pharyngeal collapsibility and compensatory airway muscle responsiveness. Traditionally, these endotypic traits have been determined in a highly controlled research laboratory setting using invasive measurement techniques (Edwards et al., 2016; Bamagoos et al., 2019b). However more recently, methods have been developed to impute endotypic traits from data accessible from routine clinical polysomnography (PSG). For example, Terrill et al. have developed a mathematical method to reliably calculate loop gain from the rise in ventilatory drive that follows an obstructive respiratory event (Terrill et al., 2015). The same group has also developed algorithms for the estimation of pharyngeal collapsibility and compensatory muscle responsiveness from the changes in ventilation and ventilatory drive seen on PSG (Sands et al., 2018). These advances pave the way for more accurate MAS prediction models unencumbered by the need for invasive laboratory studies. In a group of 93 patients with, on average, moderate OSA, greater MAS efficacy was associated with 5 endotypic traits derived using algorithms applied to clinical polysomnographic data: lower loop gain, higher arousal threshold, lower ventilatory response to arousal, moderate pharyngeal collapsibility and weaker muscle compensation (Bamagoos et al., 2019a). The association of lower loop gain and MAS response has also been confirmed in other studies (Edwards et al., 2016; Op de Beeck et al., 2021). These findings may improve prediction models for MAS response, and also raise questions for future research. For example, future studies on combination therapy with MAS plus a carbonic anhydrase inhibitor to facilitate loop gain reduction are warranted (Hedner and Zou, 2022).

Characteristics which act as direct or surrogate markers for the site of airway collapse have also been studied as predictors of response to MAS therapy. For example, the level and specific type of airway collapse observed on drug-induced sleep endoscopy (DISE) has been associated with response to MAS. Tongue-base collapse predicts a favourable response, whereas complete concentric collapse or complete latero-lateral oropharyngeal collapse are seen in those less likely to respond (Op de Beeck et al., 2019). Complete anteroposterior epiglottic collapse predicted an unfavourable response to maxillomandibular advancement surgery (Zhou et al., 2021); however MAS therapy was equally effective in patients with or without epiglottic collapse (Van de Perck et al., 2022). A posteriorly positioned tongue with a less collapsible airway is a positive predictor for MAS therapy (Marques et al., 2019). Further, certain “airflow shapes”, once again derived from routine PSG, have been used to predict the site of airway collapse and thereby response to MAS. Increased drop in airflow during respiratory events as well as a “pinched” expiratory flow shape (indicative of palatal prolapse) is associated with the poorest response to MAS therapy (Vena et al., 2020).

## 3. MAS titration technology

Traditionally, MAS devices are manually titrated under the supervision of a dentist. Various titration methods have been used, for example titrating to a percentage of maximal mandibular advancement, titrating on the basis of symptoms such as the alleviation of snoring or daytime somnolence, or titrating to an improvement in hypoxic burden which may be measured at home on overnight oximetry. Optimal titration is important to maximise the therapeutic benefits of the MAS device. An advancement of at least 50% of maximum mandibular protrusion is required to have a potential therapeutic outcome while minimising adverse side effects (Aarab et al., 2010; de Ruiter et al., 2020). However, manual titration of a MAS remains inefficient in terms of time to achieve optimal therapeutic outcomes (Sharma et al., 2013; Fleury and Lowe, 2014; Kuna, 2014). International guidelines recommend a progress diagnostic sleep study following titration to assess the efficacy of the device (Ramar et al., 2015).

Novel MAS titration techniques such as the use of a remote-controlled mandibular positioner (RCMP) to determine the therapeutic level of mandibular advancement during a single night PSG have been proposed to overcome the inefficiency barriers to MAS therapy (Pételle et al., 2002; Tsai et al., 2004; Dort et al., 2006; Remmers et al., 2013). Single night PSG titration enables reasonable prospective prediction of MAS therapy success as demonstrated by Remmers et al. (2013). However, RMCP is resource intensive, and requires the use of a sleep laboratory and trained and experienced staff.

A feedback-controlled mandibular positioner (FCMP) was recently developed to enable titration of a MAS device outside the laboratory setting (Remmers et al., 2017). The FMCP combines the use of a level 3 home sleep apnoea test (HSAT), the mechanism of the RCMP and machine learning algorithms to analyse the frequency of sleep disordered breathing and automatically titrate the mandibular advancement device accordingly to resolve sleep disordered breathing (Remmers et al., 2017). An early iteration of the FCMP demonstrated a sensitivity and specificity of 85 and 93% respectively for the prediction of therapeutic success, defined as an oxygen desaturation index (ODI) < 10/hr with the device *in situ* (Remmers et al., 2017). A subsequent iteration improved the sensitivity and specificity to 91 and 100% respectively, with 93% prediction accuracy (Mosca et al., 2022). This finding highlights the potential for future use of an auto-titrating mandibular advancement device to efficiently identify the therapeutic mandibular position. One small crossover pilot study (*n* = 10) found no difference in optimal MAS positioning using three titration methods: (1) subjective titration, (2) PSG-guided titration using a remotely controlled mandibular positioner (RCMP) and (3) DISE-assisted titration using RCMP (Kazemeini et al., 2022). Larger studies will be required to confirm the accuracy of remote titration methods compared with in-laboratory titration.

## 4. Advances in mandibular advancement splint fabrication

The European Respiratory Society recommends a custom-made titratable MAS device as preferable over non-custom devices (Ramar et al., 2015). Recent implementation of digital technologies to dentistry has transformed dental workflows for custom MAS devices (Tallarico, 2020; Alauddin et al., 2021). The use of intraoral scanners and computer aided design/computer aided manufacturing (CAD/CAM) techniques have streamlined the delivery of dental care (Tallarico, 2020; Alauddin et al., 2021). Benefits in patient preference, time savings and elimination of physical storage make digital workflows superior to conventional dental workflows (Mangano et al., 2017). Accuracy of both intraoral scanners and conventional impression methods are also comparable (Afrashtehfar et al., 2022; Hashemi et al., 2022). Device fabrication *via* CAD/CAM methods allows for the time efficient production of dental devices (van Noort, 2012). The digital technology also provides more accurate measures of tooth and jaw position, improving the quality of device fabrication, as well as facilitating superior observation and monitoring of potential dental side effects from these appliances.

Studies comparing CAD/CAM manufactured MAS devices and conventionally manufactured MAS devices are limited. One study demonstrated a significant increase of 40% in oropharyngeal airway volume in patients who used a CAD/CAM MAS device (Kerbrat et al., 2021). Similar findings were also observed in oral appliance treatment success rates of 63% (Kerbrat et al., 2021) and 80% (Vecchierini et al., 2016) in patients using CAD/CAM devices. In addition, therapy compliance and patient preference favoured the CAD/CAM oral appliances (Vecchierini et al., 2016; Kerbrat et al., 2021). The authors attributed these findings due to the differences in material, shape, and magnitude of vertical opening between devices (Vecchierini et al., 2016).

## 5. Data collection and remote monitoring

### 5.1. Adherence data

The collection of efficacy and adherence data is routine for CPAP therapy in clinical practice, and is now available for MAS therapy. The American Academy of Dental Sleep Medicine defines adequate compliance with oral appliance (OA) as a minimum of ≥80% of total sleep time per night, starting when the OA is placed in the mouth and ending when the OA is removed from the mouth, ≥5 nights per week (Radmand et al., 2021). A number of small studies have looked at objectively recorded MAS compliance over an initial 3 month period, and most have found average compliance rates to be in excess of 6 h per night (Sutherland et al., 2021b). In order to objectively assess compliance data, a temperature sensitive sensor microchip may be embedded within, or attached to, the device. When the temperature lies within a certain range (generally 31.5–39.2°C), it is inferred that the device is *in situ* within the oral cavity, and therefore in use. The device may store from 100 days to many months' worth of compliance information which is available for download *via* a base-station at the time of patient review. There are a number of models available, with sampling intervals ranging from 5 to 15 min. One study compared the accuracy of three commercially available microsensors under *in vitro* and *in vivo*, conditions, and all were found to be highly reliable (Kirshenblatt et al., 2018). Data analysis and display varies according to brand and software. One brand (Dentitrac, Braebon Ltd.) additionally collects and reports positional data (supine vs. non-supine sleep) (Sutherland et al., 2021b). Cluster analysis has identified three main MAS adherence patterns identified over 60 days of objective adherence data recording: “Consistent Users” (48.3%), “Inconsistent Users,” (32.8%) and “Non-Users” (19.0%). These usage patterns can be identified within the first 20 days of therapy, providing an early opportunity for intervention for those patients with sub-therapeutic adherence (Sutherland et al., 2021a).

Development of device-imbedded compliance chips within MAS devices opens up the possibility of remote, real-time monitoring of patient compliance. Integration of device recorded compliance data to cloud-based and patient engagement platforms may improve patient compliance to OSA therapies in some patients. For example, the use of cloud-based monitoring for CPAP therapy has demonstrated an extra hour of CPAP use per night (Hwang et al., 2018; Malhotra et al., 2018). Furthermore, the inclusion of a patient engagement tool which provides coaching and the ability for patients to view CPAP use to remote monitoring further improves device usage (Hostler et al., 2017; Hwang et al., 2018; Woehrle et al., 2018). Additionally, the adherence rate was higher compared to usual care without remote monitoring and patient engagement tools (Hostler et al., 2017; Hwang et al., 2018). While platforms for remote monitoring of MAS compliance are currently limited, integration of these platforms and telehealth modes in future oral appliances may increase the uptake of MAS devices and further improve long term adherence rates for some patients. A recent study evaluated the objective compliance with remote monitoring and therapy feedback to patients for MAS devices (Kwon et al., 2022). Similar trends were noted to that of remote monitoring and therapy feedback for CPAP in that, objective compliance to MAS therapy can be increased with remote monitoring and therapy feedback to patients (Kwon et al., 2022).

### 5.2. Biological signals

Another recent advance for MAS devices is the integration of buccal oximetry sensors. Evidence on their performance is mixed with very early success with these sensors (Rogers and Gan, 1997), but more recent work suggesting these sensors do not provide accurate oxygen saturation (SpO2) measurement and further technological work was needed to determine if it is the site, the sensors or both which is the issue (De Jong et al., 2011). In contrast, a very recent study by Nabavi et al. (2020) successfully developed a smart MAS that monitors cardiorespiratory parameters intraorally (Nabavi et al., 2020). The device comprised of a flexible hybrid wireless monitoring platform integrated within a MAS, that acquires intraoral photo-plethysmography (PPG) signals. Their results showed that the PPG signals captured intraorally are highly correlated with the conventional PPG signals received and therefore enabled the collection of heart rate (HR), respiratory rate (RR), and SpO2. The estimated values of HR, RR, and SpO2 from the intraoral PPG signals show an accuracy of over 96% with reference to PSG. Further, PPG has been combined in a single device together with positional data and breathing route (mouth vs. nose) (Nabavi and Bhadra, 2021). These developments have exciting potential clinical applications and may translate into a smart MAS device which can facilitate home-based MAS efficacy studies, home-based MAS titration studies as well as capturing data on combination therapy with MAS plus positional devices. Further, the inclusion of physiological sensors highlights the potential for the development of future FCMP devices which can more accurately auto-titrate oral appliance during sleep (Remmers et al., 2017).

Martinot et al. (2019) and Pépin et al. (2020) have shown that mandibular movements measured using midsagittal mounted magnetic sensors on the chin and the forehead, successfully differentiated obstructive and central events (Martinot et al., 2019) and when the signals were combined with machine learning could successfully discriminate controls from apnoea patients (RDI ≥ 5) with an area under the receiver operating characteristic curve (AUC-ROC) of 0.95 (Pépin et al., 2020). More recent work by the same group (Le-Dong et al., 2021) demonstrated that machine learning also enabled accurate sleep staging to be performed with an AUC for wake of 0.98, N1/N2 sleep of 0.86, N3 sleep of 0.97, and REM sleep of 0.96. Additionally, mandibular jaw movements (MJM) can be used as a surrogate measure for nocturnal respiratory effort (RE), since the slight protrusions of the mandible during sleep are reflective of respiratory drive (Martinot et al., 2022). Respiratory effort measured *via* MJM was a stronger predictor of prevalent hypertension than AHI (Martinot et al., 2022). Such sensors potentially could be embedded within MAS devices to allow capture of compliance data and cardiovascular risk profiling. European Respiratory Society guidelines have highlighted the need for rigorous validation studies for such diagnostic devices which use intelligent sensors, including the need for appropriate power calculations as well as side effect and failure rate profiling. Importantly, it is noted that since the diagnostic algorithms for such devices are not published, the sleep stages and event scoring cannot be manually reviewed or altered as they can be for level 1–4 diagnostic devices (Riha et al., 2023).

**Figure 1:**
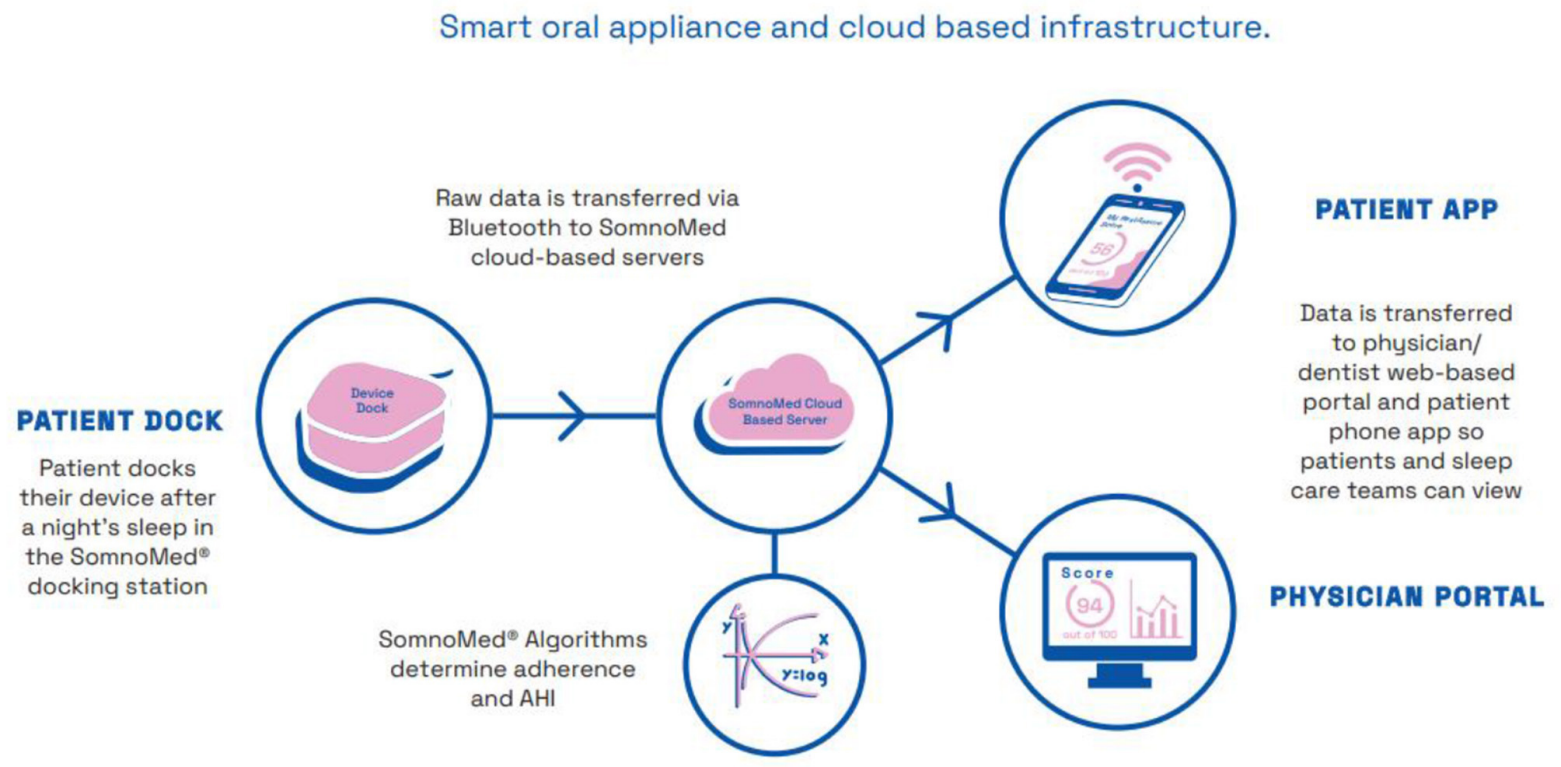
Schematic diagram illustrating mandibular movement sensor technology for the collection, storage, analysis and display of sleep data; as used in a commercial application. Image courtesy of Somnomed.

At least one manufacturer has recently recognised the potential benefits of instrumenting MAS, announcing the development of a smart oral appliance prototype hardware and software that provides a smart oral appliance with sensors to monitor efficacy and compliance^1^, see Figure 1.

## 6. Conclusion

Like many areas of medicine, OSA therapy has now entered the age of personalisation. Advances in MAS therapy discussed here will contribute to personalisation of MAS therapy at the level of patient selection, titration, improved adherence and monitoring of treatment. The use of endo-phenotypes for MAS response prediction models is likely to become more refined and thereby more accurate, allowing for targeted patient selection and combination treatment strategies. New MAS prototypes can incorporate a suite of physiological sensors that support clinical decision making with regards to titration, compliance and efficacy of the device. In particular, mandibular movement sensors have emerged which have diagnostic and treatment applications, though these require rigorous validation studies. MAS compliance monitoring and cloud-based platforms will continue to be integrated into clinical practice to improve patient engagement and compliance. These combined advances will increase the quality and safety of MAS therapy, making it available to increasing groups of patients using a targeted therapeutic approach.

## Author contributions

AM, BT, and PDC each wrote sections of the first draft of the manuscript. AM drafted the final version. All authors contributed to the scope and structure of the manuscript. All authors contributed to manuscript revisions and approved the submitted version.

## Footnotes

^1^ https://company-announcements.afr.com/asx/som/e60260ee-9367-11ec-b4cf-6eeaf7b2618c.pdf
